# Bringing Formal and Informal Reasoning Together—A New Era of Assessment?

**DOI:** 10.3389/fpsyg.2016.01097

**Published:** 2016-07-19

**Authors:** Nani Teig, Ronny Scherer

**Affiliations:** ^1^Department of Teacher Education and School Research, Faculty of Educational Sciences, University of OsloOslo, Norway; ^2^Faculty of Educational Sciences, Centre for Educational Measurement, University of OsloOslo, Norway

**Keywords:** computer-based assessment, formal reasoning, informal reasoning, interactivity, simulations

## Introduction

Scientific reasoning represents a set of skills students need to acquire in order to successfully participate in scientific practices. Hence, educational research has focused on developing and validating assessments of student learning that capture the two different components of the construct, namely formal and informal reasoning. In this opinion paper, we explain *why* we believe that it is time for a new era of scientific reasoning assessments that bring these components together, and *how* computer-based assessments (CBAs) might accomplish this.

Reasoning is a mental process that enables people to construct new representations from existing knowledge (Rips, 2004). It includes cognitive processing that is directed at finding solutions to problems by drawing conclusions based on logical rules or rational procedures (Mayer and Wittrock, 2006). When people reason, they attempt to go “beyond the information given” to create a new representation that is assumed to be true (Bruner, 1957). The process of scientific reasoning comprises formal and informal reasoning (Galotti, 1989; Kuhn, 1993). *Formal reasoning* is characterized by rules of logic and mathematics, with fixed and unchanging premises (Perkins et al., 1991; Sadler, 2004). It encompasses the ability to formulate a problem, design scientific investigations, evaluate experimental outcomes, and make causal inferences in order to form and modify theories related to the phenomenon under investigation (Zimmerman, 2007). Formal scientific reasoning can be applied not only within the context of science, but in almost every other domain of society (Han, 2013). It can be used to make informed decisions regarding everyday life problems (Amsterlaw, 2006); for example, individuals use proportional reasoning to decide the fastest way to travel from one place to another.

In *informal reasoning*, students draw inferences from uncertain premises as they ponder ill-structured, open-ended, and debatable problems without definitive solutions (Kuhn, 1991). When students reason formally, they work with the given premises in *belief mode*, which concerns arriving at true and warranted conclusions whereas informal reasoning is carried out in *design mode*, which focuses on identifying relevant premises that can be used to establish a strong argument (Bereiter and Scardamalia, 2006). Since a premise of informal reasoning is uncertain and can be questioned, its conclusion can be withdrawn in the light of new evidence (Evans, 2005). This process involves weighing the pros and cons of a particular decision (Voss et al., 1991). Learners engage in informal reasoning when they deal with *socio-scientific issues*—controversial issues that are influenced by social norms and conceptually related to science, such as whether or not to consume genetically modified food or support government's plan for a car-free city (Sadler and Zeidler, 2005).

Both types of reasoning are used to manipulate existing information and share the same goal of generating new knowledge. While formal reasoning is judged by whether or not conclusions are valid, informal reasoning is assessed based on the quality of premises and their potential for strengthening conclusions.

The manipulation of existing information in formal and informal reasoning processes can be described with dual-process theories of reasoning (Evans, 2007; Glöckner and Witteman, 2010). According to these theories, there are two distinct processing modes: Type 1 processes are autonomous and intuitive processes that do not heavily rely on individuals' working memory, whereas Type 2 processes involve using mental simulation or thought experiments to support hypothetical thinking and reflective processes that require working memory (Evans and Stanovich, 2013). An individual's first response to a problem tends to be processed automatically and refers to their past experiences and personal beliefs (i.e., Type 1 process: Evans, 2008). For example, when using formal reasoning to decide the fastest way to travel from A to B, an individual's first thought might be to take a plane since it is commonly considered the fastest means of transport. However, the individual might change his or her mind after processing all necessary information, such as the travel time to and from the airport.

Not every individual is able to progress after the first stage and produce a rational decision. Those who are confined to Type 1 processes make intuitive decisions, whereas more experienced individuals utilize Type 2 processes to construct a well-informed choice (Wu and Tsai, 2011). In the example of using informal reasoning to decide whether or not to support a government's plan for a car-free city, intuitive thought might lead individuals to support the plan based on their experiences with pollution. However, with the purpose of generating new representations, only those who can (a) elaborate on their intuitive decision with acceptable justifications; (b) address opposite arguments; and (c) think about how the plan can be further improved are utilizing Type 2 processes. In this regard, there is a strong connection between formal and informal reasoning, in which both types of reasoning share the common goal of generating new knowledge by processing available information through the dual stages.

Activity in belief mode covers a broad range of scientific practices in school science (Bereiter and Scardamalia, 2006). Outside the classroom, however, students need to make decisions regarding problems with uncertain premises by working in design mode. Teachers should have ways to assess how students improve on their existing ideas by searching beyond what they already know rather than simply making sure their ideas align with accepted theories. It is therefore important to build a scientific reasoning assessment that incorporates both formal and informal reasoning skills in order to better measure the constructs underlying scientific reasoning. In the following, we argue that these complex skills can be best assessed using computer-based testing.

## Joint assessment of formal and informal reasoning: What can computer-based testing offer?

**Figure 1:**
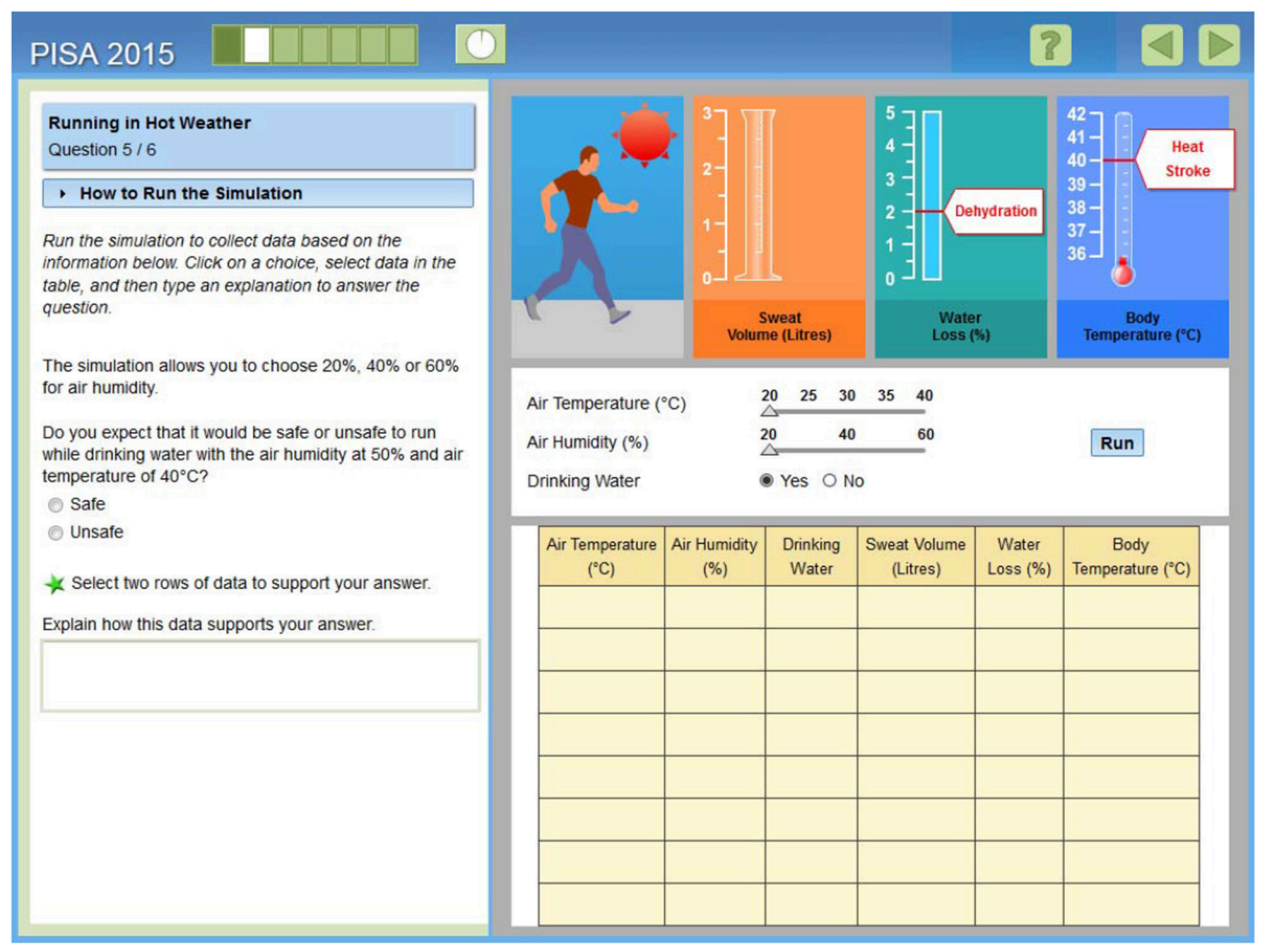
**Screenshot of the PISA (Programme for International Student Assessment) field trial item, *Running in Hot Weather***. Multivariable reasoning is required to solve the item (OECD, 2013, p. 39).

The rapid advancement of computer technology has changed the way scientific reasoning is assessed. Given that technology can offer rich reasoning activities that can be modified to serve different purposes, such as formative and summative assessment, static forms of assessment (e.g., paper-and-pencil tests) have been replaced by computer-based tests that contain dynamic and highly interactive simulations. This shift has taken place for a number of reasons: First, today's technology can deliver assessments that use multiple representations and various item formats to measure complex skills that are not easily measured in traditional paper-based testing (Quellmalz et al., 2013). Assessment of complex skills such as multivariable reasoning, in which learners disentangle the effects of independent variables on dependent variables in order to test their hypotheses, can be conducted efficiently with the use of simulations. They can run as many experiments as needed to observe how the results changes as the effects of change in input variables to test their hypotheses (see Figure 1). Second, CBAs can provide a broad range of data beyond students' mere performance on tasks. Additional information is stored in log files, including data on response times, the sequence of actions, and the specific strategies used to deal with multiple variables (Greiff et al., 2016).

Against this backdrop, we argue that CBAs have the potential to integrate approaches for assessing both formal and informal reasoning—learning outcomes that are difficult or even impossible to assess using conventional methods.

### Individual reasoning and collaborative performance

To date, CBAs have been used to comprehensively measure individual students' *formal reasoning* skills (Kuo and Wu, 2013). These assessments enable students to test their hypotheses in environments that simulate the complexity of real experiments (Greiff and Martin, 2014; Scherer, 2015). The immediate feedback such environments provide based on students' manipulation of variables can be used to develop a mental model that represents the relationship among variables. While the benefits of using CBAs for the assessment of formal reasoning skills are well-recognized, collaborative classroom discussions during group work are considered to be the main sources of information on students' *informal reasoning* skills (Driver et al., 2000). Like actual scientists, students work together to solve an authentic task through debate and argumentation (Andriessen et al., 2013). This discussion process can offer rich information on students' communication and collaboration skills; yet, it remains difficult to measure each individual's ability and contribution. CBAs offer plenty of opportunities to capture collaborative activities by keeping track of individuals' contributions to the discussion and the sequence of arguments (De Jong et al., 2012; Nihalani and Robinson, 2012). Hence, combining the assessment of formal and informal reasoning and delivering it using computer-based testing may enable us to not only investigate students' individual reasoning skills but also their performance in group discussions.

### Interactivity

Interactivity is a distinctive quality of CBA that allows individual student to demonstrate *formal reasoning* skills by interacting with a computer system (Kuo and Wu, 2013). A student participates in scientific investigations while actively exploring items that represent scientific phenomena (Quellmalz et al., 2012). During the task exploration phase, the student conducts experiments and manipulates the virtual environment in order to produce desirable outcomes. He or she engages in inquiry practices such as observing the phenomena under investigation, simulating interactive experiments by controlling variables to test their hypotheses, generating and interpreting evidence, and developing evidence-based knowledge. By using interactive and dynamic items, CBAs can examine a student's ability to coordinate complex, primarily formal reasoning skills.

To assess *informal reasoning* skills, interactive components in CBAs engage students to explore and make use of relevant information to support their arguments. When faced with a problem related to a socio-scientific issue, students can seek necessary information from a simulated website rather than using data that is already provided in the argumentation task in order to address contrasting positions and to construct a well-informed decision. Hence, CBAs provide an opportunity to assess how well students can select relevant information actively as well as their informal reasoning skills.

In addition to allowing learners to demonstrate their scientific reasoning skills, research has suggested that interactive features could improve learners' problem solving performance (e.g., Plass et al., 2009; Scherer and Tiemann, 2012). Evans and Sabry (2003) found that students who used an interactive system outperformed those using a non-interactive system. Furthermore, Quellmalz et al. (2012) showed that English Language Learners and special needs students performed better with the use of interactive, simulation-based science assessments. Interactivity is therefore considered a highly important component of building assessments of formal reasoning. Taken together, CBAs have the potential to provide stimulating, interactive environments in which students can perform both formal and informal reasoning.

### Feedback

Another feature CBAs offer in testing formal and informal reasoning skills is the ability to provide students with the necessary feedback to help them take control of their own learning. This didactic advantage can lead to better learning outcomes when feedback is given in a timely fashion and tailored to individual needs (e.g., Lopez, 2009; van der Kleij et al., 2012). Customized and instant feedback is essential for helping students understand why their responses fail to solve specific formal reasoning problems or why the information they used to support their arguments is inadequate. Students can adapt and assess their learning through gradually increasing feedback, from a brief to a more detailed scaffold (Shute, 2008). Feedback can encourage students to actively construct their own knowledge and improve their learning.

## Conclusion

On the basis of the strong conceptual connection between formal and informal reasoning, we argue that it is necessary to bring both components together for the assessment of scientific reasoning. The current developments in CBAs provide an opportunity to assess scientific reasoning in a way that reflects the complexities of formal *and* informal reasoning while also effectively measuring learning outcomes.

## Author contributions

NT drafted the paper, initiated the argumentation, and submitted the first version. RS discussed the argumentation, contributed to drafting the paper, and assisted in the submission.

### Conflict of interest statement

The authors declare that the research was conducted in the absence of any commercial or financial relationships that could be construed as a potential conflict of interest.
